# Neurotoxicity of PBDEs on the Developing Nervous System

**DOI:** 10.1289/ehp.1103907

**Published:** 2011-08-01

**Authors:** Marek Banasik, Dominika Suchecka

**Affiliations:** Institute of Public Health and Environmental Protection, Warsaw, Poland, E-mail: iphep.banasik@gmail.com

Dingemans et al. (2011) published a review article on polybrominated diphenyl ethers (PBDEs) and the developing nervous system. However, the authors summarized but failed to critically evaluate the articles cited in their review. They also did not discuss or cite literature that contradicted the studies on which they based their conclusions. For example, the U.S. Environmental Protection Agency (EPA) cosponsored an expert panel on neurodevelopmental end points, which concluded that an experimental design used in nine of the studies cited by Dingemans et al. (2011) failed to control for litter effects (Holson et al. 2008).

Although some investigators have set forth the argument that direct dosing of pups precludes the need to control for litter effects, a U.S. EPA cosponsored expert panel (Moser et al. 2005) evaluated this issue and concluded otherwise.

Regardless of whether Dingemans et al. (2011) view the studies by Holson et al. (2008) and Moser et al. (2005) as credible, the authors should have discussed them to some degree. It is understandable that because of space limitations not all studies can be included in a review. However, it was unacceptable to exclude studies that carry the weight of U.S. EPA cosponsored expert panels or other reviews that critically evaluated many of the studies cited by Dingemans et al. (2011) (e.g., Goodman 2009; Hardy et al. 2009; Williams and DeSesso 2010) and came to opposite conclusions.

Although the article by Dingemans et al. (2011) was peer-reviewed, it presents information in a selective, noncritical manner, which is best reserved for public relation pieces communicated in the non–peer-reviewed media.
